# Benefits and Toxicity of Disulfiram in Preclinical Models of Nephropathic Cystinosis

**DOI:** 10.3390/cells10123294

**Published:** 2021-11-24

**Authors:** Anna Taranta, Mohamed A. Elmonem, Francesco Bellomo, Ester De Leo, Sara Boenzi, Manoe J. Janssen, Amer Jamalpoor, Sara Cairoli, Anna Pastore, Cristiano De Stefanis, Manuela Colucci, Laura R. Rega, Isabella Giovannoni, Paola Francalanci, Lambertus P. van den Heuvel, Carlo Dionisi-Vici, Bianca M. Goffredo, Rosalinde Masereeuw, Elena Levtchenko, Francesco Emma

**Affiliations:** 1 Renal Diseases Research Unit, Genetics and Rare Diseases Research Division, Bambino Gesù Children’s Hospital, IRCCS, 00165 Rome, Italy; francesco.bellomo@opbg.net (F.B.); ester.deleo@opbg.net (E.D.L.); manuela.colucci@opbg.net (M.C.); laurarita.rega@opbg.net (L.R.R.); francesco.emma@opbg.net (F.E.); 2 Department of Clinical and Chemical Pathology, Faculty of Medicine, Cairo University, Cairo 11956, Egypt; mohamed.abdelmonem@kasralainy.edu.eg; 3 Laboratory of Pediatric Nephrology, Department of Development and Regeneration, KU Leuven, 3000 Leuven, Belgium; Bert.vandenHeuvel@radboudumc.nl (L.P.v.d.H.); elena.levtchenko@uzleuven.be (E.L.); 4 Laboratory of Metabolic Biochemistry Unit, Department of Pediatric Medicine, Bambino Gesù Children’s Hospital, IRCCS, 00165 Rome, Italy; sara.boenzi@opbg.net (S.B.); sara.cairoli@opbg.net (S.C.); carlo.dionisivici@opbg.net (C.D.-V.); bianca.goffredo@opbg.net (B.M.G.); 5 Division of Pharmacology, Utrecht Institute for Pharmaceutical Sciences, Utrecht University, 3584 CG Utrecht, The Netherlands; m.j.janssen1@uu.nl (M.J.J.); a.jamalpoor@uu.nl (A.J.); r.masereeuw@uu.nl (R.M.); 6 Genetics and Rare Diseases Research Division, Bambino Gesù Children’s Hospital, IRCCS, 00165 Rome, Italy; anna.pastore@opbg.net; 7 Histology-Core Facility, Bambino Gesù Children’s Hospital, IRCCS, 00165 Rome, Italy; cristiano.destefanis@opbg.net; 8 Department of Pathology, Bambino Gesù Children’s Hospital, IRCCS, 00165 Rome, Italy; isabella.giovannoni@opbg.net (I.G.); paola.francalanci@opbg.net (P.F.); 9 Department of Pediatric Nephrology, Radboud University Medical Center, 6525 GA Nijmegen, The Netherlands; 10 Division of Pediatric Nephrology, Department of Pediatrics, University Hospitals Leuven, 3000 Leuven, Belgium; 11 Division of Nephrology, Department of Pediatric Subspecialities, Bambino Gesù Children’s Hospital, IRCSS, 00165 Rome, Italy

**Keywords:** cystinosis, disulfiram, mice, zebrafish

## Abstract

Nephropathic cystinosis is a rare disease caused by mutations of the CTNS gene that encodes for cystinosin, a lysosomal cystine/H+ symporter. The disease is characterized by early-onset chronic kidney failure and progressive development of extra-renal complications related to cystine accumulation in all tissues. At the cellular level, several alterations have been demonstrated, including enhanced apoptosis, altered autophagy, defective intracellular trafficking, and cell oxidation, among others. Current therapy with cysteamine only partially reverts some of these changes, highlighting the need to develop additional treatments. Among compounds that were identified in a previous drug-repositioning study, disulfiram (DSF) was selected for in vivo studies. The cystine depleting and anti-apoptotic properties of DSF were confirmed by secondary in vitro assays and after treating *Ctns^-/-^* mice with 200 mg/kg/day of DSF for 3 months. However, at this dosage, growth impairment was observed. Long-term treatment with a lower dose (100 mg/kg/day) did not inhibit growth, but failed to reduce cystine accumulation, caused premature death, and did not prevent the development of renal lesions. In addition, DSF also caused adverse effects in cystinotic zebrafish larvae. DSF toxicity was significantly more pronounced in *Ctns^-/-^* mice and zebrafish compared to wild-type animals, suggesting higher cell toxicity of DSF in cystinotic cells.

## 1. Introduction

Nephropathic cystinosis (NC) is an inherited metabolic disease secondary to mutations in the CTNS gene, which encodes for cystinosin, a cystine proton symporter allowing efflux of cystine from lysosomes [1]. In patients with NC, cystine progressively accumulates in nearly all tissues. Symptoms begin with renal Fanconi syndrome in the first year of life, followed by cystine crystal depositions in the cornea [2]. In time, patients develop other symptoms, including hypothyroidism, pancreatic insufficiency, gonadal failure, poor growth, myopathy, cholestatic liver disease, and central and peripheral nervous system involvement [3]. Kidney damage is characterized in part by increased apoptosis of proximal tubular cells in mice [4], confirming previous in vitro studies showing that cystinotic cells are more sensitive to apoptotic stimuli [5,6].

Cysteamine was approved for the treatment of NC in the 1990s, and allows cystine clearance from lysosomes through the formation of a mixed cysteine–cysteamine disulfide that can exit lysosomes through the PQLC2 transporter [7]. Cysteamine significantly improves NC, but does not cure the disease. Despite adequate treatment, the majority of patients progress to end-stage kidney disease in the second or third decade of life [8]. 

In order to identify new molecules that can improve the treatment of patients with NC, two drug screenings were performed using conditionally immortalized human cystinotic proximal tubule cells [9]. A high throughput screening based on cell cystine concentration identified 24 compounds that reduced cystine content by >50%. Similarly, a high content screening identified 27 compounds that decreased apoptosis (caspase-3/7 positivity) by >40% [9]. We combined results from these two screenings, and identified disulfiram (DSF) as a potential treatment for cystinosis. Herein, we report the results of in vitro and in vivo studies assessing the efficacy of DSF in cystinotic human proximal tubule epithelial cells, mice, and zebrafish. Our results confirm the cystine-depleting and anti-apoptotic effects of DSF, but show significant toxicity that is enhanced in cystinotic animal models.

## 2. Materials and Methods

### 2.1. Cell Culture

In vitro assays were performed using conditionally immortalized proximal tubule epithelial cells (ciPTECs) obtained from the urine of a patient with nephropathic cystinosis or from healthy controls that have been described elsewhere [10] and were provided to us by Radboud University Medical Center (Nijmegen, The Netherlands). Growth media included DMEM-F12 medium supplemented with 10% FBS, Penicillin (100 U/mL)/Streptomycin (0.1 mg/mL), ITS (5 µg/mL Insulin, 5 µg/mL Transferrin, 5 ng/mL Selenium), hydrocortisone (36 ng/mL), EGF (10 ng/mL), and tri-iodothyronine (40 pg/mL). Cells were grown at 33 °C for proliferation and 37 °C for 7 days to allow differentiation. Medium, FBS and Penicillin/Streptomycin were supplied by Gibco (Thermo Fisher Scientific, Waltham, MA, USA). All other reagents were from Sigma-Aldrich (Merck, Darmstadt, Germany).

### 2.2. Quantitative Determination of Cystine in Cells

Cystinotic ciPTECs were seeded at a density of 5 × 10^4^ cells/well in 48-well plates. After 48 h, cells grown at 33 °C were treated with different concentrations of DSF (from 0.1 to 100 µM) and cysteamine. After 24 h, cells were washed twice in PBS and lysed in 75 µL of 10 mM N-ethylmaleimide (NEM) with five freezing/thawing cycles. Cell lysates were precipitated with 75 µL of 10% 5-sulfosalicylic acid (SSA) and left overnight at 4 °C. Plates were then centrifuged at 3900× *g* for 15 min at 4 °C. Supernatant (25 µL) was analyzed by reverse-phase high-performance liquid chromatography and fluorescence detection (HPLC-FLD) for thiol measurements [11]. The remaining samples were used to measure protein content after adding 50 µL of 0.1 M NaOH and BCA reagent (Bio Rad Laboratories, Hercules, CA, USA).

### 2.3. Measurement of Apoptosis in Cells

Cystinotic ciPTECs were seeded at a density of 4 × 10^3^ cells/well in 384-well plates coated with poly-D-lysine (Perkin Elmer, Waltham, MA, USA). After 48 h, cells were pre-treated with different concentrations of DSF for one hour and apoptosis was induced with Fas-ligand (0.5 µg/mL) and cycloheximide (10 µg/ml) for 5 h. ciPTECs were then incubated with 4 µM of cellEvent probe (Invitrogen life technologies, Carlsbad, CA, USA) for 30 min. This reagent is a cell permeant dye that emits fluorescence when cleaved by caspases-3/7. For the analysis, cells were fixed in 4% paraformaldehyde and nuclei were stained with Hoechst 33258. Cells were imaged with the automated Opera system (Perkin Elmer, Beaconsfield, UK). Apoptotic cells were quantified as positive nuclei/total number of cells. We performed 16 replicas per each treatment; 250 cells/well were analyzed.

### 2.4. Cell Viability Assay

Cystinotic and wild-type ciPTECs were seeded at a density of 55 × 10^3^ cells/cm^2^ and grown at 33 °C for 24 h and 37 °C for 7 days. Cells were then treated for 24 h with L-cysteine (1.8 mM), N-acetyl cysteine (1.8 mM), DSF (different concentrations; see below), or a combination of the above. Cell viability was evaluated using the PrestoBlue Cell Viability Reagent (Thermo Fisher Scientific) according to the manufacturer’s instructions. 

### 2.5. Redox Status

Cystinotic ciPTECs were seeded and grown as described above. After treatment with 10 µM DSF, cells were incubated with CellROX Green Reagent (Thermo Fisher Scientific) for 30 min at room temperature and reactive oxygen species (ROS) were measured by live cell flow cytometry. GSH and GSSG levels were analyzed as described by Jamalpoor et al., who have performed extensive metabolomic investigations in the same cell conditions [12].

### 2.6. Tandem Mass Spectrometry 

L-cystine (416 µM at pH 7.4) and DSF (200 µM in DMSO) were stirred overnight at RT. Tandem mass spectrometry analyses were carried out on a 4000-QTRAP mass spectrometer (ABSciex, Toronto, ON, Canada), equipped with a Turbo Ion Spray Source operating in positive ion mode with a needle potential of 5500 V. The flow rate of mobile phase was set at 150 µL/min and infused into the spectrometer using an Agilent 1290 infinity pump (Agilent Technologies Inc., Wilmington, DE, USA). Instrument setting and calibration were performed with 10 µM DSF and 10 µM cystine solutions. The analysis was conducted to evaluate the formation of hybrid disulfide molecule of 269 m/z.

### 2.7. Studies on Cystinotic Mice

Ctns knockout mice (C57BL/6 background) were kindly provided by Dr. Corinne Antignac [13]. Animal care and experimental procedures were conducted in accordance with the European 2010/63/EU directive on the protection of animals used for scientific purposes, and authorized by the Italian Ministry of Health (authorization number 230/2015-PR).

Drugs were mixed with standard 4RF21 diet and prepared in pellets (Mucedola, Settimo Milanese, Italy). Concentrations of drug in pellets were calculated based on the average daily food intake (approximately 3 g/day) and body weight (approximately 22 g) of female mice, to administer estimated doses of 50, 100, and 200 mg/kg of body weight/day of DSF. Female mice started treatment at 2 months of age. Animal length and weight were measured monthly. Every two months, mice were acclimatized in metabolic cages for 24 h, and their urine was collected for 24 h. Blood samples were collected at sacrifice. Analyzed parameters included urine volume, electrolytes, glucose, proteins, BUN, and creatinine. Measurements were performed by the Appia Laboratory (Rome, Italy). In addition, low-molecular weight proteinuria was estimated using the Clara cell 16 protein (CC16) as a marker (Biomatik Corporation, Kitchener, ON, Canada). Power analysis was performed to calculate the minimum number of mice per treatment arm.

### 2.8. Quantitative Determination of Diethyldithiocarbamate (DDC) and Cystine in Tissues

For DDC quantitative determination, 10 mg of tissue samples were sonicated in 100 μL NaCl 0.9% solution. The supernatant (50 μL) was extracted with 200 µL of acetonitrile, vortexed and centrifuged at 18,000× *g* for 9 min. A solution of DDC was prepared by dissolving in methanol to make up a standard solution of 1 mg/mL. 

For cystine quantitative determination, tissue samples were sonicated in the presence of 10 mM NEM. Homogenates were centrifuged at 1000× *g* for 5 min and supernatants were mixed with 10% SSA (3:1 volume ratio), incubated at 4 °C for 60 min, and centrifuged at 20,000× *g* for 15 min. The supernatants (50 μL) were spiked with 50 μL of the internal standard solution (Cystine d6). The mixture was then extracted with 200 µL of acetonitrile, vortexed and centrifuged at 18,000× *g* for 9 min. Protein concentrations were measured on the first supernatant using Bio-Rad Protein Assay Reagent Kit (Bio-Rad Laboratories Inc.), following the manufacturer’s protocol. All chemicals were of analytical grade and were obtained from Sigma-Aldrich (St. Louis, MO, USA).

Liquid chromatography/mass spectrometry analysis was performed using a UHPLC Agilent 1290 Infinity II 6470 (Agilent Technologies Inc.) equipped with an ESI-JET-STREAM source operating in positive ion (ESI+) mode. The MassHunter Workstation software (Agilent Technologies Inc.) was used for data analysis. InfinityLab Poroshell 120 HILIC 1.9 μm 100 × 2.1 mm (Agilent Technologies Inc.) were used as separation columns. A full validation assay was performed, including selectivity, specificity, linearity, limits of quantification, accuracy, precision, matrix effects, recovery, and stability.

### 2.9. Measurement of Apoptosis in Tissue

Immunohistochemistry was performed on 2 µm thick sections obtained from formalin-fixed tissue embedded in paraffin. After dewaxing and rehydrating, heat-induced epitope retrieval was performed by boiling the slides with sodium citrate (pH 6) (Dako, Glostrup, Denmark). Endogenous peroxidase was blocked with 3% hydrogen peroxide and then with 5% BSA. Sections were incubated overnight at 4 °C with rabbit monoclonal (9661S) to Cleaved Caspase-3 (Cell Signaling Technology, Danvers, MA, USA), diluted 1:400. Detection of the primary antibody was performed by using the appropriate secondary biotinylated antibody (K8024) (ready to use) (Dako, Carpinteria, CA, USA) and the peroxidase DAB kit (Dako, Carpinteria, CA, USA). Counterstaining was performed with hematoxylin solution Gill2.

### 2.10. Zebrafish Assays

Experiments were performed on wild-type and cystinotic zebrafish larvae that have already been characterized [14]. Adult fish were raised at 28.5 °C, on a 14/10 h light/dark cycle under standard aquaculture conditions [15]. Fertilized wild-type or cystinotic embryos (20–30 per well) were transferred to 6-well plates containing 5 mL of clean egg water (Instant Ocean Sea Salts 60 μg/mL + methylene blue 0.5 ppm). DSF was dissolved directly in the egg water at the specified concentrations. Embryos were incubated at 28.5 °C in the dark and the medium was refreshed daily. Wells were cleaned from debris daily and dead embryos were removed and counted. Larvae viability was monitored for the first 96 h post-fertilization (hpf). Hatching rates were calculated in surviving embryos at 48, 72, and 96 hpf. Final deformity rates were evaluated at 96 hpf. Animal care and experimental procedures were conducted in accordance with the ethical committee guidelines for laboratory animal experimentation at KU Leuven, in accordance with the European 2010/63/EU directive on the protection of animals used for scientific purposes. 

For cystine quantitative determination, homogenates of surviving zebrafish larvae were assessed using liquid chromatography tandem mass spectrometry. Groups of 30–60 larvae for each condition were homogenized by sonication in 200 μL of 5 mM NEM in 0.1 M PBS. Then, 100 μL of 12% SSA was added to each homogenate and samples were centrifuged for 10 min at 4 °C at 12,000× *g*. Supernatants were used for cystine measurements and stored at −80 °C. Pellets for protein measurements were dissolved overnight at 4 °C in 300 μL of 0.1 M NaOH, and stored at −80 °C.

### 2.11. Statistical Analysis

Categorical data are represented as counts and percentages. Continuous normal data are expressed as mean ± standard error of the mean. Continuous data that do not follow a normal distribution are expressed as median value and interquartile range. Normality of data was tested with the D’Agostino–Pearson test. The Chi-squared test was used to compare hatching and deformity rates. Student’s *t*-test, ANOVA followed by Bonferroni’s post-hoc correction, and the Mann–Whitney U-test were used as appropriate. Survival was estimated by the Kaplan–Meier method and assessed by the Log-Rank test. All *p*-values are two-sided and considered significant for *p* < 0.05. Statistical analyses were performed using the GraphPad Prism 6 software (San Diego, CA, USA).

## 3. Results

### 3.1. In Vitro DSF Studies 

At the end of two drug screenings [9] that used cystine accumulation and apoptosis as read-outs, we identified six compounds that showed positive effects in both assays. Among these, we selected DSF for future tests, based on its pharmacological properties and known safety profile. 

**Figure 1:**
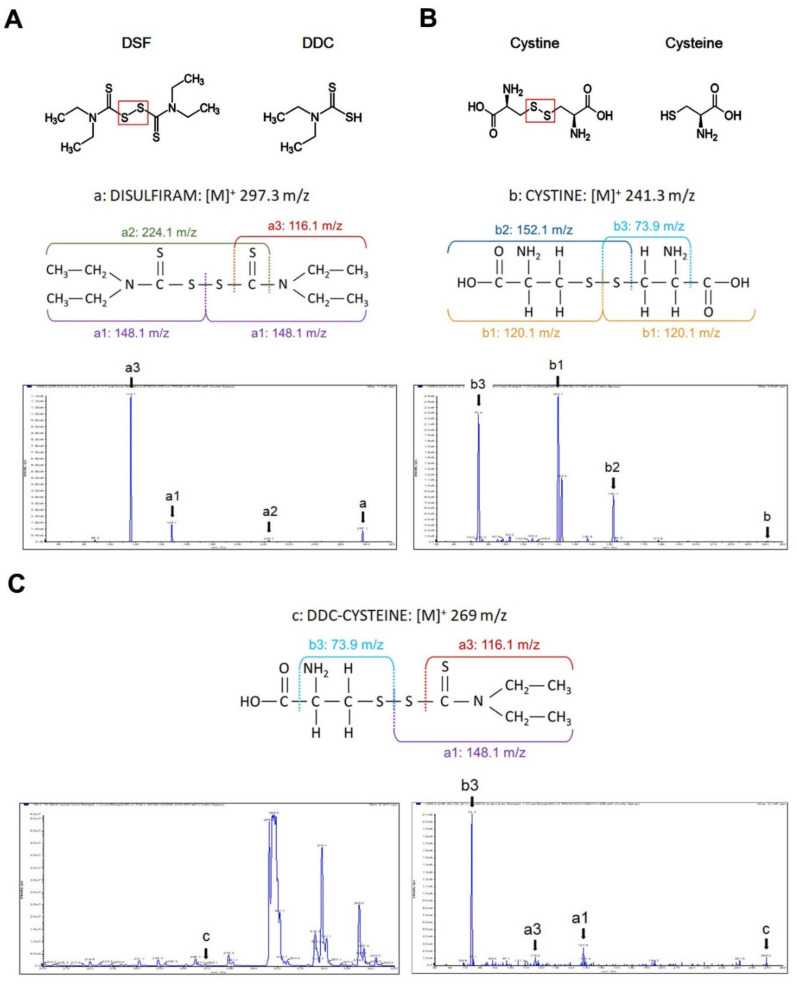
MS/MS determination of DSF, cystine and mixed-disulfides. (**A**) Chemical structures of DSF and diethyldithiocarbamate (DDC). The chromatogram shows peaks corresponding to DSF (a), half molecule of DSF (a1) and two molecules obtained by breaking the carbon-sulfur bond (a2 and a3). (**B**) Chemical structures of cystine and cysteine. The chromatogram shows peaks corresponding to cystine (b), half molecule of cystine (b1), and fragments obtained after breaking carbon-sulfur (b2) and carbon–carbon bonds (b3). (**C**) Mixed disulfides. The chromatogram on the left panel shows a peak indicating the formation of a mixed-disulfide with a calculated mass–charge ratio (m/z) of 269 (c). The right panel shows the product ion scan for the 269 m/z peak, identifying three fragments: the 148 m/z peak corresponds to half molecules of DSF (a1), the 116 m/z peak is produced by thiol-ester bond cleavage of DSF (a3), and the 74 m/z peak corresponds to cystine (b3).

Unlike cysteamine, which is the current standard of care for cystinosis, DSF is a disulfide compound composed of two S-methyl-N,N-diethyldithiocarbamate (DDC), lacking free sulfhydryl residues (Figure 1A,B).

We hypothesized that under physiologic conditions, cystine and DSF are in equilibrium with their reduced form, allowing the formation of mixed disulfides. To this end, we co-incubated liquid solutions of DSF and cystine for 14 h at room temperature and analyzed them by tandem mass spectrometry. As illustrated in Figure 1C, several mixed disulfides formed, indicating that the cystine-lowering effect of DSF is likely mediated by the formation of mixed disulfides that can bypass the non-functioning cystinosin transporter, similarly to what has been described for cysteamine.

**Figure 2:**
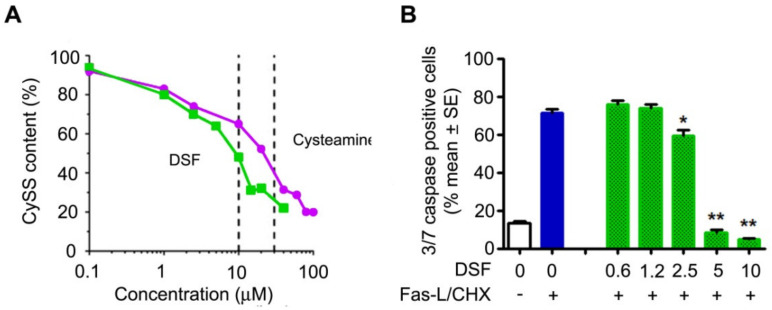
Cystine-depleting and anti-apoptotic proprieties of DSF. (**A**) Comparison of dose–response curves. Cystine (CySS) accumulation was measured after treatment for 24 h with the indicated concentrations of DSF and cysteamine in cystinotic ciPTECs. Vertical dashed lines indicate IC50 for DSF (9 µM) and cysteamine (22 µM). Data represent 4 independent experiments. (**B**) Anti-apoptotic dose response of DSF. The white and blue columns indicate spontaneous or induced apoptosis with Fas-Ligand (Fas-L) and cycloheximide (CHX) in cystinotic ciPTECs, respectively. The green columns indicate the treatment with DSF (0.6–10 µM) in cells exposed to apoptotic stimuli. Data represent 3 independent experiments. * *p* < 0.005 and ** *p* < 0.0005 vs. blue column.

We then performed dose-response experiments to identify the lowest concentration of DSF that prevented cystine accumulation and apoptosis in cystinotic ciPTEC and compared the effect with cysteamine. As shown in Figure 2A, DSF lowered cystine slightly better (IC50: 9 µM) than cysteamine (IC50: 22 µM). DSF also prevented apoptosis at concentrations ≥2.5 µM (Figure 2B).

### 3.2. In Vivo Studies: Murine Treatment with High DSF Dose 

**Figure 3:**
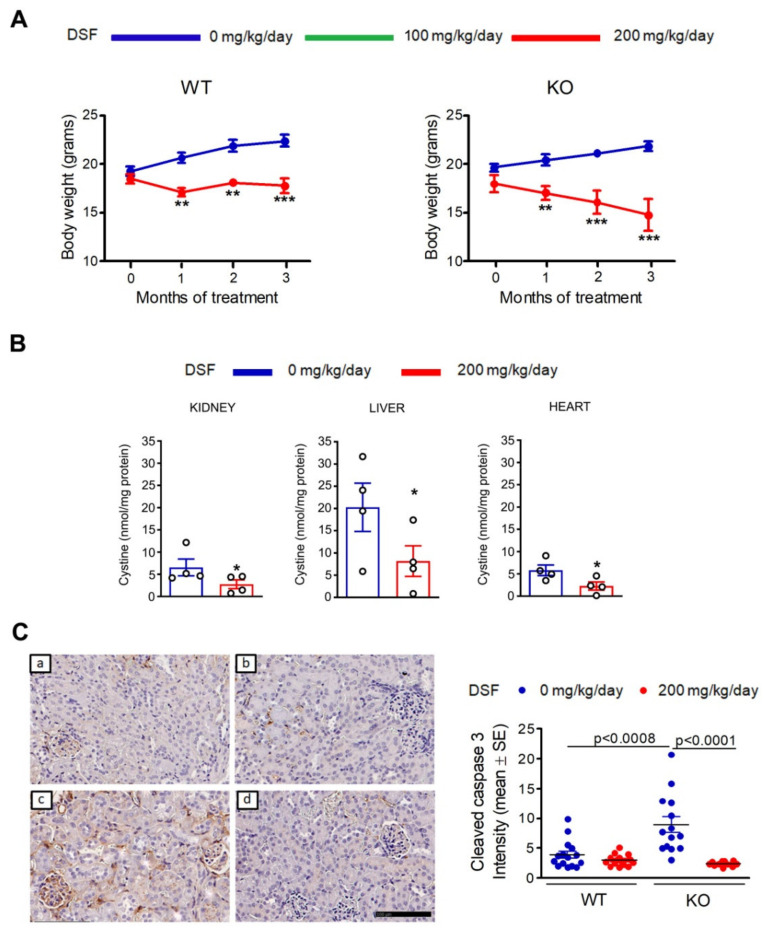
Short-term treatment with high DSF dose. Two-month-old mice were fed on standard or DSF-supplemented diet at an estimated dose of 200 mg/kg of body weight/day. (**A**) Body weight in animals fed on standard or DSF-supplemented diets for three months. Data are shown as mean ± SEM; *n* = 9 WT and 4 KO mice; ** *p* < 0.005, *** *p* < 0.0005 compared to untreated animals. (**B**) Cystine content in kidneys, liver, and heart of KO animals sacrificed at 5 months of age (*n* = 4 mice per group). Values are indicated as mean ± SEM; * *p* < 0.05 compared to untreated mice. (**C**) Representative immunohistochemistry images stained with anti-cleaved caspase-3 antibodies in kidneys obtained from WT (**a**,**b**) and KO (**c**,**d**) mice that were fed on standard (**a**,**c**) or DSF-supplemented (**b**,**d**) diets. Scale bar: 100 µm. The graph shows the mean cleaved caspase-3 intensity measured on five separate slides per animal (*n* = 3 mice per group).

**Figure 4:**
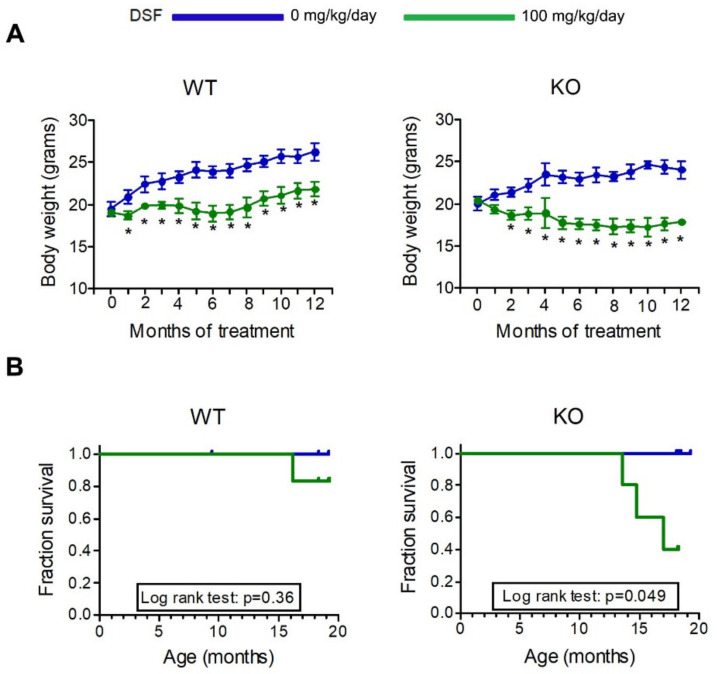
Long-term treatment with low DSF dose. Two-month-old mice were fed on standard or DSF-supplemented diets at an estimated dose of 100 mg/kg/day for 16 months. (**A**) Body weight; data are shown as mean ± SEM; *n* = 5 mice per group; * *p* < 0.05 compared to untreated animals. (**B**) Kaplan–Meier survival curves in the same animals.

Based on the above results, we treated *Ctns* knockout (KO) mice with DSF. KO and wild-type (WT) animals received DSF in food from the age of 2 months. Two different concentrations of DSF were initially used, corresponding to an estimated daily dose of 100 (low dose) or 200 (high dose) mg/kg (see methods). We rapidly observed in both WT and KO animals significant growth impairment after DSF treatment, especially at the highest dose (Figure 3A and Figure 4A). We therefore decided to sacrifice animals treated with the 200 mg/kg/day dose for three months (i.e., at 5 months of age). Biochemical and histopathological parameters were evaluated to measure kidneys and liver function. 

From the kidney standpoint, untreated KO mice at 5 months of age had more glycosuria compared to WT animals, and developed marked low-molecular weight proteinuria (LMWP) (Table 1). Treatment with high-dose DSF decreased glucose excretion, increased calciuria, and had no impact on low-molecular weight proteinuria (Table 1). Serum creatinine and blood urea nitrogen levels were unchanged (data not shown).

Evaluation of liver function tests revealed increased plasma alkaline phosphatase levels in DSF-treated animals compared to untreated animals, which did not exceed the normal range (Table S1). All other studied markers were unaltered (Table S1). By standard liver histology techniques, we did not observe abnormal changes, including fibrosis, steatosis, inflammation, or signs of hepatocellular damage (data not shown).

After sacrifice, we measured the impact of DSF on cystine accumulation and apoptosis. As shown in Figure 3B, cystine content was decreased in the kidneys, liver, and heart of DSF-treated KO mice. Kidney histology did not show pathological changes, including tubular atrophy, interstitial fibrosis, mesangial expansion, or glomerular damage in both DSF-treated and untreated KO animals (data not shown). However, an increased number of apoptotic cells was observed in untreated cystinotic kidneys (Figure 3C), but not in kidneys from KO animals treated with high-dose DSF (Figure 3C), confirming the in vitro observations (Figure 2B).

### 3.3. In Vivo Studies: Murine Treatment with Low DSF Dose 

Animals receiving 100 mg/kg/day DSF dose were monitored for 18 months. Urine parameters were available every 2 months, until the age of 1 year. Intermediate results are illustrated in Figure S1; results at 12 months are detailed in Table 2. At 1 year of age, KO mice had markedly more albuminuria, low-molecular weight proteinuria, and diuresis compared to WT mice. DSF treatment failed to improve urinary parameters. 

On average, body weight of mice treated with DSF was lower compared to untreated animals (Figure 4A). After the age of 12 months, mice were followed until death or sacrificed at 18 months. As shown in Figure 4B, treatment with DSF decreased survival only in KO animals (*p* = 0.049).

During the initial dose-testing assays, we also tested a pilot cohort of five KO mice treated with a DSF dose of ~50 mg/kg/day. Of these, three animals died between 16 and 18 months. Since most animals treated with the 100 mg/kg/day dose had died before the age of 18 months, we pooled together the data of four surviving KO animals treated with either 50 or 100 mg/kg/day of DSF. These results are shown in Table S2 and Supplementary Figure S2. We observed no significant differences between treated and untreated animals. In particular, we failed to observe a decrease in tissue cystine content, unlike what we observed at 5 months in animals treated with the 200 mg/kg/day dose. This was not related to decreased food intake, which was monitored, and is further substantiated by high DDC levels in tissues obtained from treated animals (Figure S2). 

### 3.4. In Vivo Studies: Zebrafish Embryos and Larvae 

**Figure 5:**
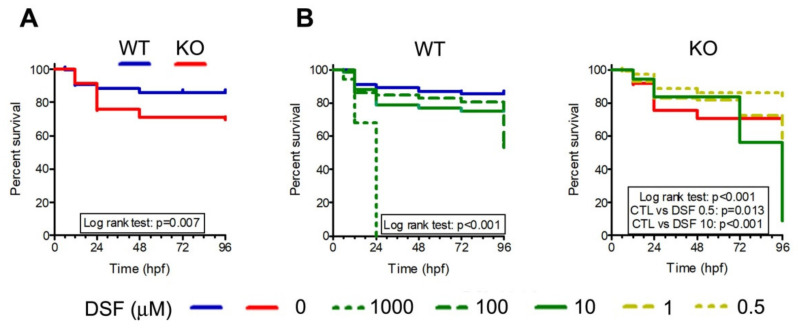
Effect of DSF on survival in wild-type (WT) and cystinotic (KO) zebrafish embryos and larvae. (**A**) Survival curves of untreated KO (*n* = 82) and WT (*n* = 126) embryos and larvae. (**B**) Survival curves of WT and KO embryos treated with different DSF concentrations as indicated in the figure legend. All curves are obtained from at least 100 larvae. Survival was monitored for 96 h post-fertilization (hpf).

In parallel to murine studies, we performed assays on cystinotic (KO) zebrafish to assess DSF safety. WT and KO zebrafish embryos were treated within 1 h after fertilization with different concentrations of DSF. Hatching rates were monitored at 48, 72, and 96 h post-fertilization (hpf) and dysmorphic features were analyzed at 96 hpf. Embryos were monitored for mortality rates at different time points (6, 12, 24, 48, 72, and 96 hpf). As shown in Figure 5A, the mortality rate was higher in untreated KO larvae than in untreated WT larvae (*p* < 0.007). Larvae mortality increased very rapidly with increasing doses of DSF in KO fishes (Figure 5B). The 50% lethal concentration of DSF (LD50) was 146 µM in WT embryos and 2 µM in KO embryos (96 hpf).

**Figure 6:**
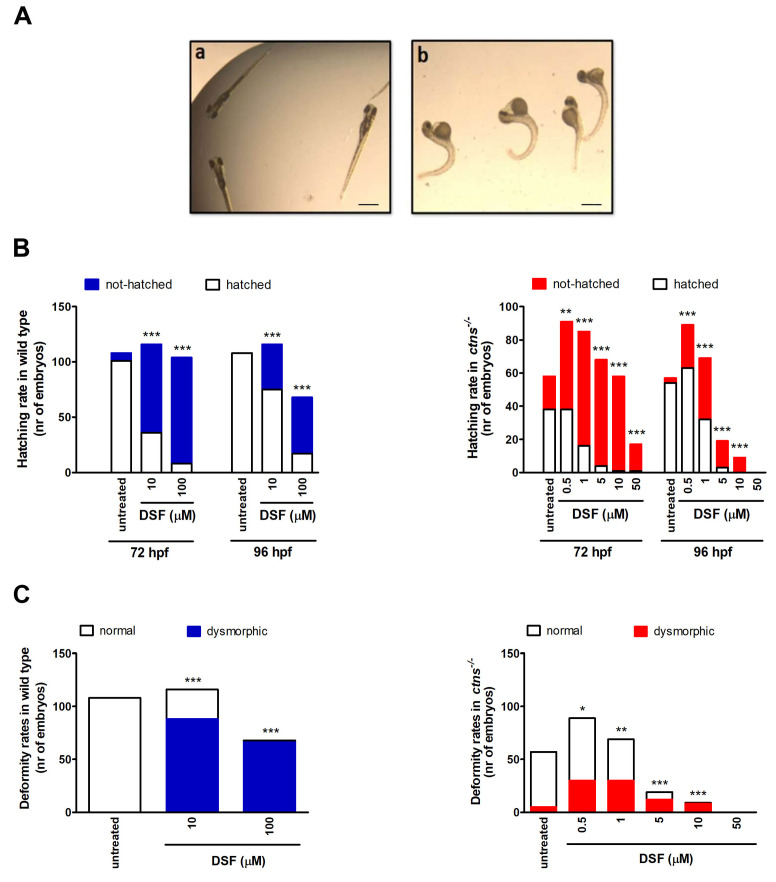
Effect of DSF on hatching and deformity rate in wild-type (WT) and cystinotic (KO) zebrafish embryos and larvae. (**A**) Representative microscopy image of WT (a) and KO (b) zebrafish embryos. Scale bar: 1 mm. (**B**,**C**) Hatching and deformity rates in embryos and larvae treated with different doses of DSF (WT: *n* = 116 and 104 at 10 and 100 μM, respectively; KO: *n* = 91, 85, 68, 58, and 17, at 0.5, 1, 5, 10, and 50 μM, respectively). Deformity rates were evaluated after 96 hpf. * *p* < 0.05, ** *p* < 0.01, *** *p* < 0.001 compared to untreated embryos or larvae.

Similarly, hatching was delayed and larval malformations were significantly more frequent in zebrafish treated with DSF (Figure 6A–C). These effects were observed at very low DSF concentrations (0.5 µM) in KO larvae, and were remarkably less pronounced in WT larvae, indicating increased sensitivity to DSF in cystinotic larvae. The average cystine levels after exposure of KO larvae to 1 µM DSF for 120 h was 2.4 ± 0.87 and 3.7 ± 0.54 nM cystine/mg protein in untreated (*n* = 11) and treated (*n* = 3) larval homogenates, respectively. At these DSF concentrations, the drug had no apparent cystine lowering effect. At higher concentrations, DSF was too toxic. Taken together, the above results suggest that DSF has a specific toxicity in cystinotic zebrafish.

### 3.5. N-Acetyl Cysteine Can Rescue Disulfiram Toxicity in Cystinotic Cells

**Figure 7:**
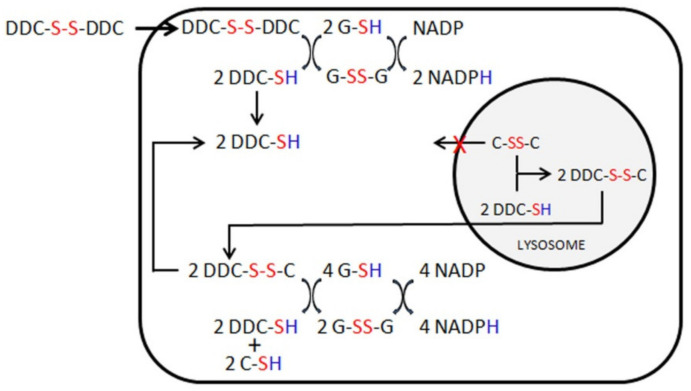
A hypothetical model illustrating the effects of DSF in cystinotic cells. DSF (DDC-S-S-DDC) is a disulfide that is reduced to diethyldithiocarbamate (DDC) in the cytosol, consuming GSH and other free thiols. Consequently, cells are more exposed to free radicals, causing oxidative cell damage and death. Similarly to cysteamine, reduced DDC can react with cystine (C-SS-C) in lysosomes, forming a mixed disulfide (DDC-S-S-C) that allows clearance of cystine.

Based on our in vivo results, we hypothesized that DSF reacts with free cellular thiols, oxidizing the cells (Figure 7).

**Figure 8:**
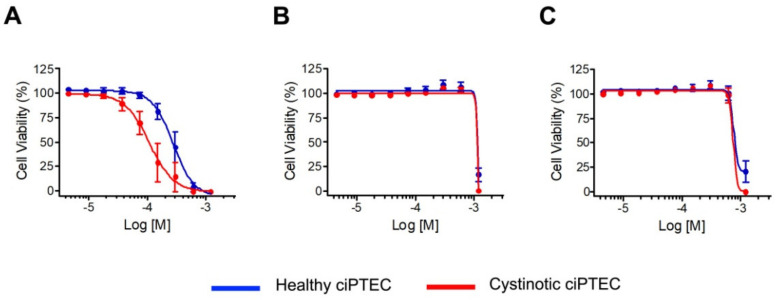
DSF toxicity in cystinotic cells. (**A**) Cell viability was measured in healthy and cystinotic ciPTEC after incubation with increasing DSF doses (from 4.5 µM to 1.2 mM). (**B**,**C**) The same experiment was repeated after incubation with L-cysteine (1.8 mM) or N-acetyl cysteine (1.8 mM) for 24 h.

We therefore measured in vitro the redox status in cystinotic ciPTEC treated with 10 µM DSF for 24 h. This dose was shown to decrease cystine accumulation and apoptosis in vitro (Figure 2). Under these conditions, the levels of reactive oxygen species (ROS) remained stable (0.99 ± 0.003 vs. 0.79 ± 0.07; *p* = 0.09, in untreated and treated cells, respectively), while the GSH/GSSG ratio decreased by more than 50% (127 ± 3 vs. 59 ± 10; *p* < 0.02 in untreated and treated cells, respectively). In addition, we also confirmed higher cell mortality in cystinotic cells exposed to DSF (LD50: 107 µM in cystinotic cells vs. 275 µM in wild-type cells) (Figure 8A). Cystinotic-specific DSF toxicity was completely abolished when cells were cultured with L-Cysteine or with N-acetyl cysteine (Figure 8B,C).

## 4. Discussion

Cystinosis is a severe metabolic disease causing cystine accumulation in most tissues. Currently, the disease is treated with cysteamine. This treatment improves clinical outcome, but progression of chronic kidney failure cannot be prevented and other long-term complications still develop in many patients. Our study aimed at identifying new treatments for cystinosis. To this end, we screened a compound library using two characteristic phenotypes of cystinotic cells, namely cystine accumulation and high propensity to undergo apoptosis [9]. Among the compounds that we identified, we selected DSF as a potential molecule for drug repositioning.

DSF is a synthetic molecule that was produced by the German chemist M. Grodzki in 1881 [16]. It was initially used in the rubber industry and was subsequently investigated for clinical use. In the early 1940s, DSF was proposed for treating scabies and worm infections, based on its copper chelating activity. These clinical assays revealed the potential use of DSF to treat alcohol abuse [16]. DSF diffuses rapidly in most tissues [17], where it interacts with sulfhydryl residues of proteins [18,19]. In particular, DSF inhibits acetaldehyde dehydrogenase in the liver, and renders subjects intolerant to alcohol. Recently, DSF has been reported to have in vitro and in vivo anti-inflammatory properties [20], which may be relevant in cystinosis, since cystine crystals cause inflammation [21,22].

Our data show that DSF decreases cystine accumulation and increases resistance of cystinotic cells to apoptosis in vitro. Apoptosis of cystinotic cells has been attributed to cysteinylation of protein kinase C delta [23] and/or to oxidative damage of mitochondria [24].

DSF appeared particularly interesting because it has been used for more than 50 years in humans for various clinical purposes. It has a relatively good safety profile in subjects treated for alcohol abuse, and has a very low cost, which would be extremely valuable in low-income countries. To our knowledge, DSF has never been used in cystinosis. Unfortunately, our results show that prolonged exposure to DSF is toxic to mice, and that toxicity is markedly increased in cystinotic animals. Higher toxicity was also confirmed in zebrafish harboring a nonsense mutation in exon 8 of the *ctns* orthologue gene. In this model, we observed increased lethality and severe developmental abnormalities at early embryonic stages. We did not observe a cystine-depleting effect, unlike what has been reported with cysteamine [14], probably because larvae died at cystine-lowering concentrations. Although disappointing, these results demonstrate the importance of a thorough drug evaluation, even when using a drug repositioning approach based on molecules where extensive clinical data are available. Of note, we cannot exclude that an intermittent treatment would have produced positive results with fewer side effects.

The murine cystinotic model that was used in these studies reproduces only in part the human disease. Similarly to humans, mice accumulate large amounts of intracellular cystine, but kidney disease develops later, and many long-term complications of cystinosis are not observed. From the renal standpoint, the phenotype is characterized by progressive development of low molecular weight proteinuria from three months of age, followed by albuminuria around 6 months of age, and chronic renal failure around one year of age. Glycosuria has a biphasic evolution. It increases from 3 to 6 months and regress around the age of 10 months. This trend has been observed in different laboratories using the same mouse model and has no clear explanation. It is remarkable that glycosuria regressed faster in animals treated with DSF, which may be related to the hypoglycemic effect of DSF. This has been shown in obese mice treated with similar doses of DSF [25]. Remarkably, the authors of this latter study observed that DSF increases energy expenditure and causes weight loss [26], which may explain, at least in part, poor animal growth in our study.

The reasons for the observed increased mortality in DSF-treated KO mice remains an open question. The zebrafish data point to a specific toxic effect of DSF on cystinotic larvae. In other cell models, oxidative stress has been proposed to be the main mechanism of DSF-mediated cell toxicity [27,28,29]. Cystinotic cells have relative glutathione deficiency and produce more ROS when exposed to oxidative stress [30,31,32]. Cell oxidation can also cause apoptosis. The anti-oxidative proprieties of cysteamine may explain the anti-apoptotic effects of this drug [24,33]. In addition, antioxidant treatment has been shown to protect proximal tubular cells in the same cystinotic mouse model [34,35]. Further supporting the role of pathogenic oxidation in cystinosis, we observed that N-acetylcysteine and L-cysteine restore cell viability of cystinotic cells treated with DSF. 

We also observed the formation of mixed disulfides of DDC with cysteine, similarly to what has been described with cysteamine. This finding supports the cystine-lowering effect of DSF, but also proves that DSF is reduced to DDC in cells, which can severely oxidize cells, as illustrated in Figure 6. Conversely, DSF may have positive effects. In particular, it may prevent apoptosis by forming mixed disulfides with caspase-3, which inhibits the enzymatic activation of the apoptosis cascade [36,37]. Our results show, however, that the net balance between these opposite effects is unfavorable in cystinotic cells. 

## Figures and Tables

**Table 1:** Urinary parameters in WT and KO mice treated with high DSF dose for three months.

		WT		KO	
Urine Tests	Measure Unit	Untreated	DSF 200	Untreated	DSF 200
Albumin	µg/mg Creatinine	5.05 [3.49–12.1]	7.66 [6.59–11.1]	8.58 [5.02–9.79]	20.7 [10.5–76.3]
Glucose	mg/mg Creatinine	0.29 [0.26–1.02]	0.34 [0.24–0.78]	7.59 [4.41–16.2] ^§^	0.40 [0.28–1.66] *
LMWP	µg/mg Creatinine	38.2 [13.8–39.5]	37.7 [10.2–69.1]	157 [75.1–447] ^§^	258 [79.6–474]
Calcium	mg/mg Creatinine	0.12 [0.09–0.17]	0.12 [0.09–0.18]	0.21 [0.15–0.26]	1.17 [0.52–3.36] *
Phosphate	mg/mg Creatinine	0.60 [0.14–1.66]	1.51 [0.15–2.88]	0.66 [0.15–1.85]	2.58 [0.99–6.43]

Data are represented as median [interquartile range]; *n* = 4 mice per group. Low molecular weight proteins (LMWP), 200 mg/kg/day DSF dose (DSF 200). ^§^ *p* < 0.05 untreated WT mice vs. untreated KO mice; * *p* < 0.05 untreated KO mice vs. treated KO mice.

**Table 2:** Urinary parameters in WT and KO mice at 12 months of age, after 10 months of treatment with DSF.

		WT		KO	
Urine Tests	Measure Unit	Untreated	DSF 100	Untreated	DSF 100
Albumin	µg/mg Creatinine	5.05 [4.26–6.40]	11.5 [5.68–13.8]	15.1 [14.4–36.2] ^§^	19.0 [17.9–36.8] *
Glucose	mg/mg Creatinine	0.18 [0.08–0.84]	0.42 [0.19–1.12]	2.71 [1.34–3.85] ^§^	9.00 [4.21–29.4] *
LMWP	µg/mg Creatinine	16.4 [5.30–26.6]	35.4 [26.8–63.2]	1939 [985–4353] ^§^	5758 [1434–13972] *
Calcium	mg/mg Creatinine	0.30 [0.25–0.35]	0.26 [0.22–0.35]	0.32 [0.30–0.57]	0.42 [0.21–0.54]
Phosphate	mg/mg Creatinine	2.08 [0.70–2.77]	1.74 [0.17–2.50]	2.93 [1.47–3.56]	2.62 [1.03–3.53]
Diuresis	ml	1.20 [0.75–2.10]	1.75 [1.62–2.00]	3.05 [2.32–3.25] ^§^	3.00 [1.60–3.25]

Data are represented as median [interquartile range]; *n* = 5 mice per group. Low molecular weight proteins (LMWP), 100 mg/kg/day DSF dose (DSF 100). ^§^ *p* < 0.05 untreated WT mice vs. untreated KO mice; * *p* < 0.05 untreated WT mice vs. treated KO mice.

## Supplementary Materials

The following are available online at https://www.mdpi.com/article/10.3390/cells10123294/s1, Figure S1: Urine parameters in mice: long-term treatment with low dose DSF. Wild-type (dashed line) and cystinotic (continuous line) mice were fed on standard (blue line) or DSF-supplemented- diet (100 mg/kg/day, green line). Urine was collected every two months. Data are reported as median values; *n* = 5 mice per group. Figure S2: Cystine and diethyldithiocarbamate (DDC) in 18-month-old mice treated with low DSF doses. WT and KO mice were fed on standard diet or DSF supplemented diets at 50 (square green dots) and 100 (round green dots) mg/kg/day. Cystine (A) and DDC (B) content in kidneys, liver and heart. Each dot represents the mean cystine values measured on two aliquots of tissue per animal; *n* = 4 or 5 per group. * *p* < 0.05, ** *p* < 0.01, *** *p* < 0.001. Table S1: Hepatotoxicity parameters measured in plasma of WT and KO mice treated with disulfiram (DSF) for three months. Table S2: Hepatotoxicity and urinary parameters in WT and KO mice at 18 months of age, after 16 months of treatment with disulfiram (DSF).
