# Supplementary material for: Benefits and Toxicity of Disulfiram in Preclinical Models of Nephropathic Cystinosis
Source: Cells. 2021 Nov 24;10(12):3294. doi: 10.3390/cells10123294 (PMC8699074; doi:10.3390/cells10123294)
Supplement: Supplementary file 1 [file cells-10-03294-s001.zip › cells-1442276-supplementary.pdf]

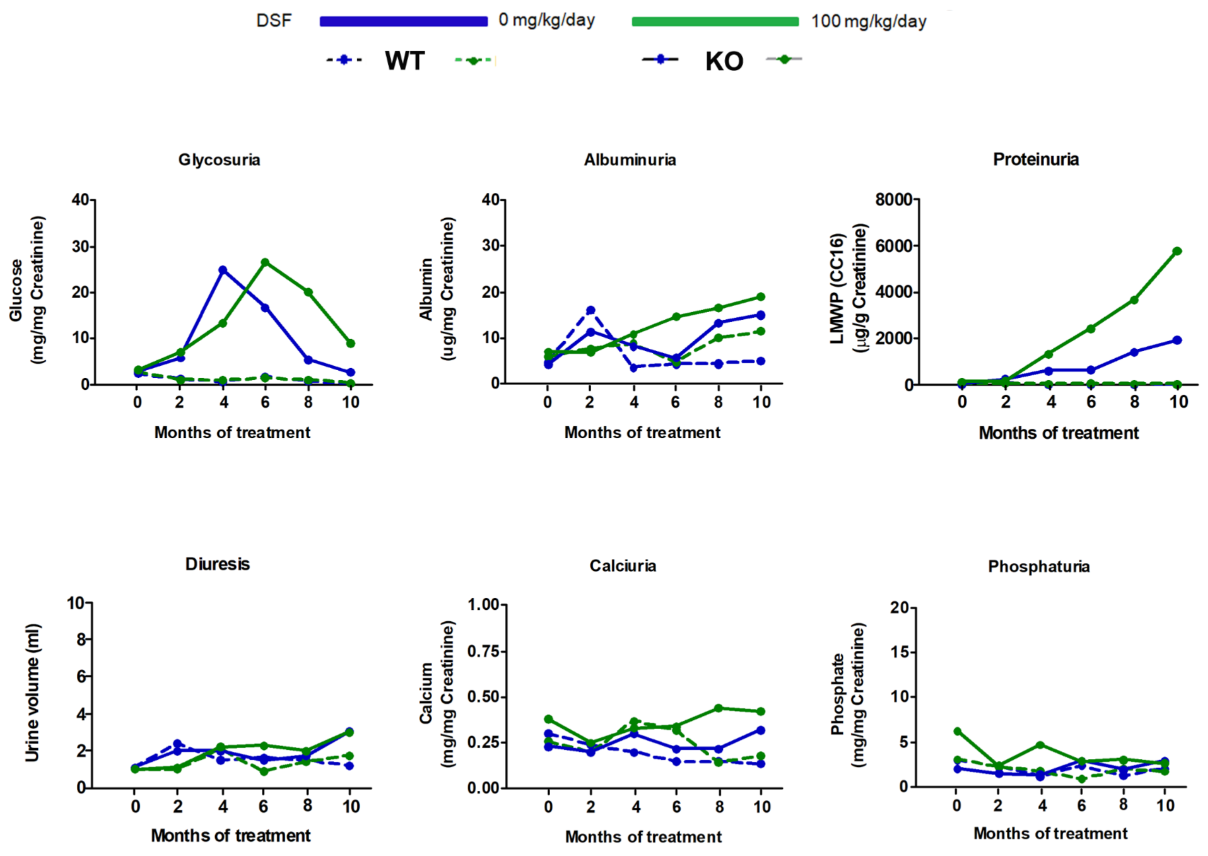

Figure S1. Urine parameters in mice: long-term treatment with low dose DSF. Wild-type (dashed line) and cystinotic (continuous line) mice were fed on standard (blue line) or DSF-supplemented- diet (100 mg/kg/day, green line). Urine was collected every two months. Data are reported as median values; n= 5 mice per group.

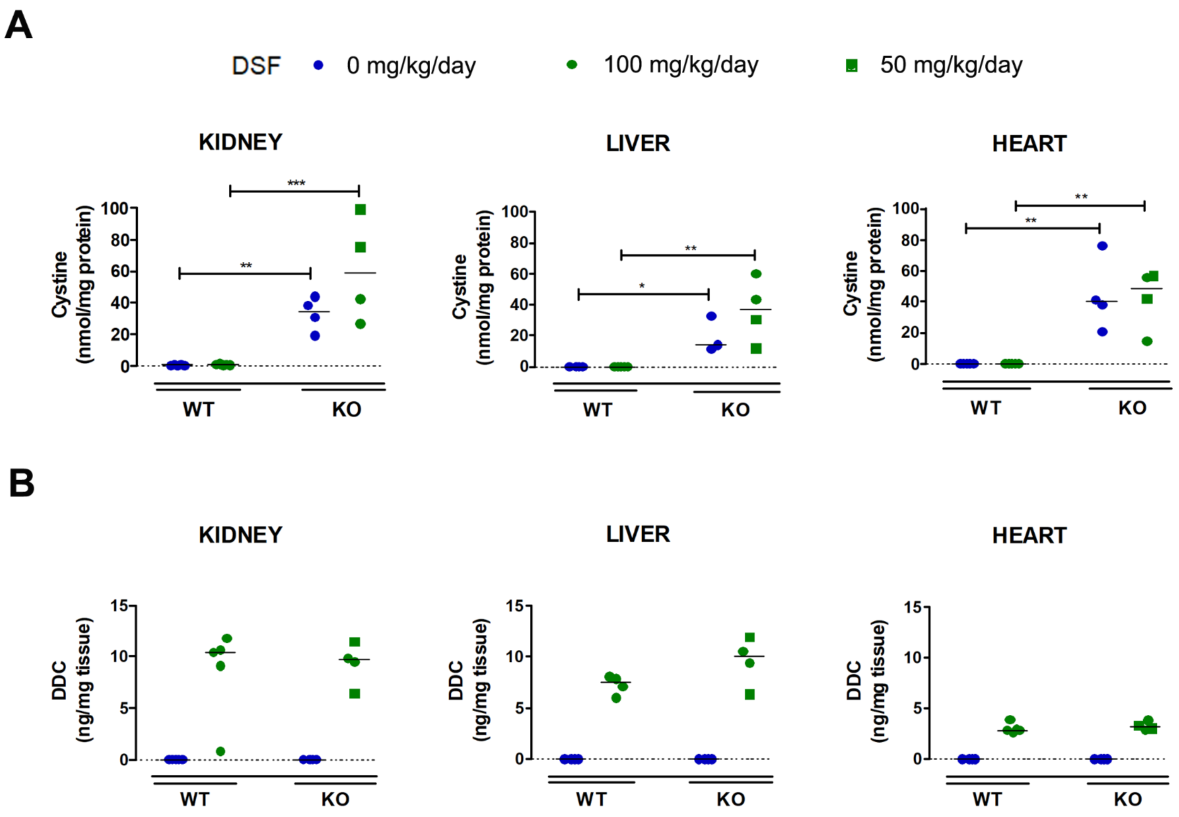

Figure S2. Cystine and diethyldithiocarbamate (DDC) in 18-month-old mice treated with low DSF doses. WT and KO mice were fed on standard diet or DSF supplemented diets at 50 (square green dots) and 100 (round green dots) mg/kg/day. Cystine (A) and DDC (B) content in kidneys, liver and heart. Each dot represents the mean cystine values measured on two aliquots of tissue per animal; n= 4 or 5 per group. *p<0.05, **p<0.01, ***p <0.001.

Table S1. Hepatotoxicity parameters measured in plasma of WT and KO mice treated with DSF for three months

		WT		KO	
Blood tests	Normal range	Untreated	DSF 200	Untreated	DSF 200
AST	54-269 IU/L	109 [89.6-149]	220 [74.9-322]	144 [98.2-337]	295 [200-386]
ALT	26-77 IU/L	38.5 [28.1-107]	34.2 [27.4-318]	45.9 [33.3-368]	45.6 [29.5-56.2]
ALP	45-222 IU/L	67.4 [62.5-81.5]	92. 0 [83.9-96.5]*	69.0 [62.1-75.6]	111 [87.3-129]*
Bilirubin	0.1-0.9 mg/dL	0.80 [0.71-0.81]	0.74 [0.57-0.76]	0.75 [0.69-0.77]	0.80 [0.65-0.91]
γGT	6-8 IU/L	15.6 [12.4-16.2]	14.1 [12.3-16.5]	14.5 [12.6-16.9]	15.0 [13.7-17.0]

Data are represented as median [interquartile range]; n=4 mice per group. Aspartate aminotransferase (AST), Alanine aminotransferase (ALT), Alkaline Phosphatase (ALP), gamma-glutamyltransferase (γGT), 200 mg/kg/day DSF dose (DSF 200). *$p<0.05$ Untreated mice vs treated mice.

Table S2. Hepatotoxicity and urinary parameters in WT and KO mice at 18 months of age, after 16 months of treatment with disulfiram (DSF)

		**WT**		**KO**	
		Untreated	DSF 100	Untreated	DSF 50-100
Blood tests	Normal range				
AST	54-269 IU/L	180 [116-314]	325 [209-376]	75.6 [43.9-131]	206 [137-258]
ALT	26-77 IU/L	56.0 [39.2-62.0]	72.0 [48.3-182]	45.0 [32.1-58.7]	53.0 [33.0-63.0]
ALP	45-222 IU/L	92.6 [87.0-215]	145 [88.1-195]	157 [122-185]	154 [104-231]
Bilirubin	0.1-0.9 mg/dL	0.22 [0.17-0.38]	0.36 [0.20-0.48]	0.33 [0.24-0.66]	0.56 [0.19-0.63]
γGT	6-8 IU/L	5.00 [4.50-6.00]	4.00 [3.00-5.50]	6.00 [4.50-6.00]	6.00 [4.50-8.50]
Urine tests	Measure Unit				
Albumin	μg/mg Creatinine	0.18 [0.11-2.61]	3.41 [2.51-7.58]	17.2 [13.1-138]*	19.3 [13.9-92.6]
Glucose	mg/mg Creatinine	0.84 [0.19-1.08]	1.16 [0.76-1.24]	0.25 [0.21-0.28]	0.35 [0.34-0.37] §
Calcium	mg/mg Creatinine	0.34 [0.25-0.38]	0.25 [0.20-0.34]	0.27 [0.23-0.45]	0.59 [0.29-0.78]
Phosphate	mg/mg Creatinine	2.04 [0.54-2.20]	1.67 [0.58-2.91]	1.97 [0.92-4.06]	1.95 [1.37-2.70]
Diuresis	ml	2.40 [1.50-3.90]	1.60 [1.27-3.30]	4.50 [3.62-5.75]	2.90 [2.57-4.12]

Data are represented as median [interquartile range]; n=4-5 mice per group. Aspartate aminotransferase (AST), Alanine aminotransferase (ALT), Alkaline Phosphatase (ALP), gamma-glutamyltransferase (γGT), 100 and 50 mg/kg/day DSF doses (DSF 50-100).
*p<0.05 untreated WT mice vs untreated KO mice

[§]$p<0.05$ untreated KO mice vs treated KO mice
